# Biotelemetry reveals migratory behaviour of large catfish in the Xingu River, Eastern Amazon

**DOI:** 10.1038/s41598-019-44869-x

**Published:** 2019-06-11

**Authors:** Lisiane Hahn, Eduardo G. Martins, Leonardo D. Nunes, Luís Fernando da Câmara, Leonardo S. Machado, Domingos Garrone-Neto

**Affiliations:** 1 Neotropical Consultoria Ambiental, Rua Cesário Rossetto, 182, Passo Fundo, RS 99074-210 Brazil; 2 University of Northern British Columbia (UNBC), Ecosystem Science and Management Program, 3333 University Way, Prince George, BC V2N 4Z9 Canada; 3 UNESP – Univ Estadual Paulista, Campus Experimental de Registro, Avenida Nelson Brihi Badur, 430, Registro, SP 11900-000 Brazil

**Keywords:** Animal migration, Animal behaviour

## Abstract

We used a combination of radio and acoustic telemetry to assess the movements of large catfish (Pimelodidae) in the Xingu River, a clearwater tributary of the Amazon River in Brazil. A total of 121 *Phractocephalus hemioliopterus* and 61 *Pseudoplatystoma punctifer* were tagged for monitoring within a 685 km segment, including the Belo Monte Hydroelectric Complex (BMHC), between February 2013 and July 2015. Long distance upstream movements were detected for *P*. *hemioliopterus* (up to 347 km) and for *P*. *punctifer* (up to 164 km) mainly during the transition between dry season and the rising water period. Both species moved through a long segment of rapids previously thought to function as barriers to migration. Several individuals exhibited long-distance bidirectional movements. Some tagged fish never left the release zone, indicating mortality, tag loss or resident individuals, which would characterize partial migration. The findings show evidence of migratory behaviour for large catfish within the Xingu River, emphasizing the influence of the hydrologic cycle on their movements. As part of the study area has become partially dewatered due to the BMHC, findings support the need of adequate management strategies to allow the movements of large catfish between spawning and feeding sites in the Xingu River.

## Introduction

The ecology, migration and conservation of large catfish (Pimelodidae) in the Amazon basin has been the subject of studies for decades, highlighting the economic importance and the use of long migratory corridors^1,2^. However, most available data on the Amazonian catfish are restricted to the species of the genus *Brachyplatystoma*, which perform the longest freshwater migration in the world (up to 5,800 km), from the mouth of the Amazon River to the headwaters of its main whitewater tributaries in Peru, Bolivia and Colombia^3,4^. The movements of other pimelodids in clearwater rivers that drain to the Lower Amazon, such as the Xingu and Tapajós, remain poorly understood. The lack of data about basic aspects of biology, ecology and even taxonomy of Amazon catfish species in clearwater rivers severely restrict the establishment of effective management strategies for their conservation. Such lack of information is of especial concern due to the rapid development of dams in Amazonian rivers.

Currently, the Amazon basin is considered the final frontier for hydroelectric development in South America, with the damming of important tributaries of the Amazon River already concluded, planned or under construction^5,6^. The increase in the number of dams in the Amazon basin has led to several discussions on how they may affect large migratory catfish, especially due to their economic and social importance for riverine communities and the function played by these large fish as predators in the Amazon ecosystem^1,7–9^. While the number of hydropower projects is increasing in the Amazon basin, many questions on the migratory behaviour and the role of rapids and waterfalls as natural barriers to migration of large catfish remain poorly understood. Filling this information gap before damming is particularly important because fish passage systems have been suggested as the main alternative for maintaining connectivity of critical habitats for large catfish in the Amazon basin. However, recent studies have indicated that fish passage systems as a management tool are ineffective or even deleterious for many Neotropical fish species^10–15^. Furthermore, the design of fish passage systems usually does not take in account the natural variation in motion and navigation capacity among species, populations and individuals^16^.

**Figure 1:**
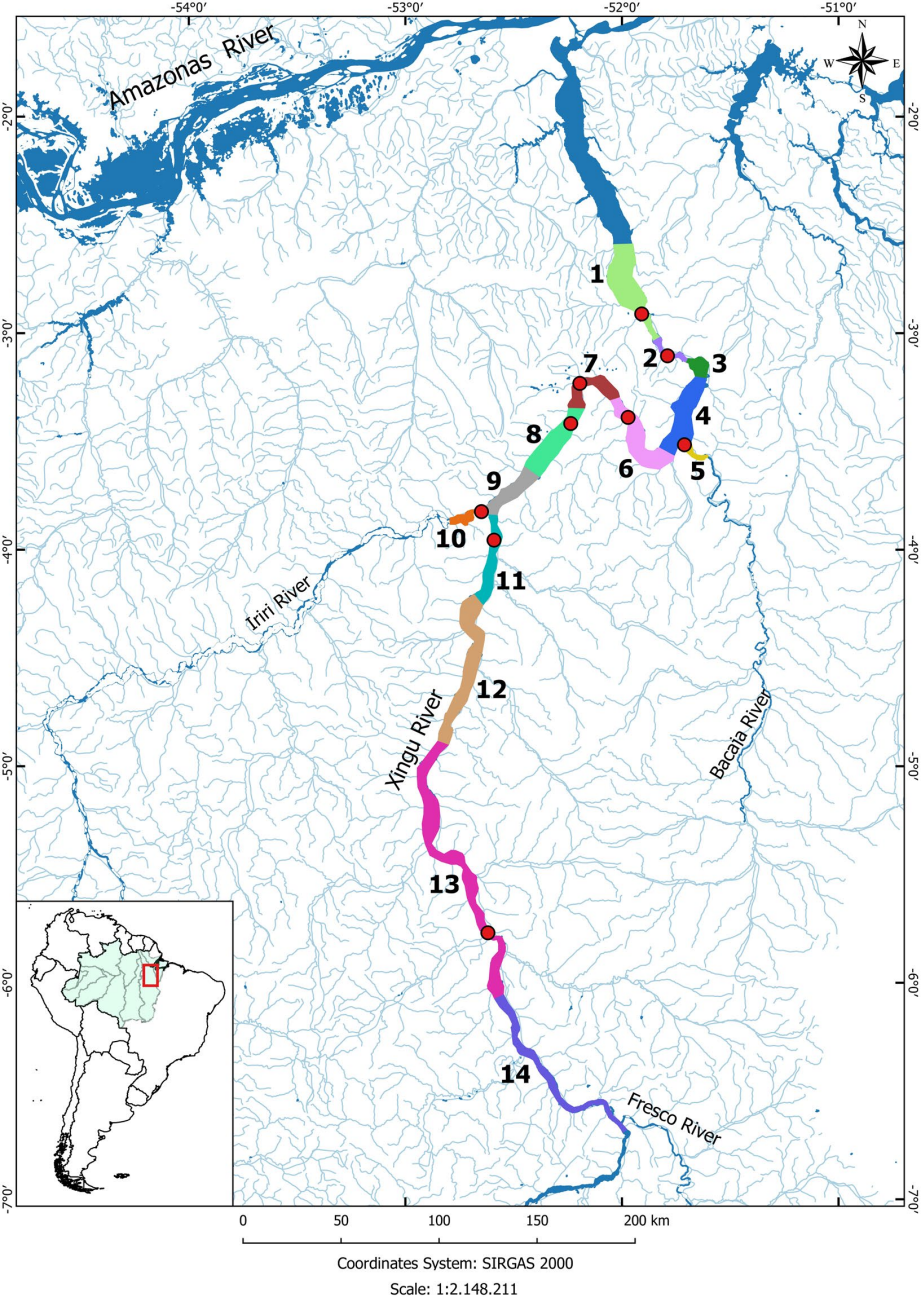
Study area in the Xingu River and some tributaries. Fixed monitoring stations with acoustic and radio telemetry (red circles). Numbers indicate zones and colors denote the zone limits. Radio tracking by boat and aircraft was conducted throughout the study area to complement the passive monitoring. Map generated in Quantum GIS ver 2.18. QGIS Development Team (2016). QGIS Geographic Information System. Open Source Geospatial Foundation Project. http://qgis.osgeo.org.

Most of the existing dams in the Amazon are concentrated in rivers which drain from ancient granitic shield such as Tocantins, Tapajós and Xingu^17^. The Xingu River, the largest clearwater tributary of the Amazon River, extends over 1,900 km from its headwaters, in the crystalline bedrock of the Brazilian Shield, to its confluence with the Amazon River, approximately 420 km from the Atlantic Ocean. With predominantly sandy and rocky bottoms and a watershed that drains 520,292 km^2^ ^18^, the Xingu River includes a section of high fish diversity and endemism known as the Big Bend (locally named “Volta Grande do Xingu”; zones 3 to 7 on Fig. 1), in the State of Pará (about 03°20′S, 52°00′W)^17,19,20^ in Brazil (Fig. 1). The Big Bend drops 90 meters in elevation over 130 km and contain numerous rapids and waterfalls flowing through a labyrinth of braided channels over fractured basement rocks^21^.

The Belo Monte Hydroelectric Complex (BMHC), with an installed capacity of 11,233 MW, was recently constructed within the Big Bend. The BMHC has a run-of-the-river design with two powerhouses: the Pimental, a supplementary power house with six generating units of Bulbo type (233 MW) located in the uppermost section of the Big Bend (zone 6, Fig. 1) where the only fish passage system of the BMHC was installed; and the “Belo Monte”, located downstream of the Big Bend (zone 2, Fig. 1), with 18 units of Francis type turbines and an installed generation capacity of 11,000 MW. Two reservoirs were formed by the BMHC: one was built in the main channel of the Xingu River (Main Reservoir) and another one (Intermediate Reservoir) was built as a diversion out of the river channel, receiving water from the main reservoir and diverting it to the Belo Monte powerhouse^20,22^. The construction of BMHC started in June 2011 and was completed in 2016. Since the completion, the Big Bend has become partially dewatered throughout the flood cycle^23^ and the current flow estimates that remain in this section are 17–25% of the historic mean total annual discharge^17^. The reduction in the natural flow can reduce dramatically the ability of migratory fish species to move longitudinally throughout the Big Bend and reach the upstream stretches of the Xingu River and its tributaries.

In this study, we used a combination of radio and acoustic telemetry to assess the movements of two species of large catfish, *Phractocephalus hemioliopterus* (redtail catfish or pirarara in Portuguese) and *Pseudoplatystoma punctifer* (tiger sorubim or surubim in Portuguese), in the Xingu River during the construction of BMHC and before the total impoundment of the river (February 2013 to July 2015). The objectives of this study were to: (i) detect and characterize the downstream and upstream movements of *P*. *hemioliopterus* and *P*. *punctifer*; (ii) determinate whether they could move through a series of rapids and falls, and (iii) determine the biotic and abiotic correlates to the initiation and rate of these movements. We hypothesized that both species are able to overcome natural obstacles (e.g. rapids) historically considered as barriers to migration in the Big Bend, and that the long-distance movements of these fish are influenced by the hydrologic cycle.

## Results

We tagged a total of 182 individuals (Table 1) with combined acoustic and radio transmitters (CART): 121 *Phractocephalus hemioliopterus* and 61 *Pseudoplatystoma punctifer*, which were detected along 14 zones in the study area (Fig. 1).

**Table 1:** Standard length (Ls) and weight (W) of tagged *P. hemioliopterus* and *P. punctifer* in the Xingu River.

Species	Standard Length (cm)			Total Weight (kg)		
	Mean (±1SD)	Min	Max	Mean (±1SD)	Min	Max
*P. hemioliopterus*	61.4 (±17.2)	32	115	6.9 (±6.8)	1.7	35.6
*P. punctifer*	55.5 (±7.3)	40	76	2.3 (±1.1)	1.2	6.4

### *Phractocephalus hemioliopterus*

**Figure 2:**
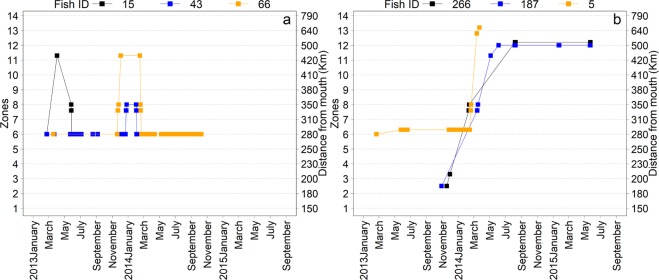
Examples of bidirectional movements (**a**) and long-distance movements (**b**) of individuals of *P. hemioliopterus* in the study area.

We detected a total of 92 (76% of 121 tagged) *P*. *hemioliopterus* after release. Forty fish (43.5% of 92) were always detected in the same zone where they were released. Individual detections from 10 (10.9%) of these fish always occurred within 2 km, suggesting that the fish were either dead or the transmitters were shed from the fish. Forty-two fish (45.7%) made unidirectional movements between zones, with three (3.3%) and 39 (42.4%) fish moving downstream and upstream of the release zone, respectively. Ten fish (10.9%) made bidirectional movements between zones, with one fish (1.1%) first moving downstream and then upstream across zones; and nine fish (9.8%) first moving upstream and then downstream across zones. In all these bidirectional cases, the fish returned to the same zone from where they had departed (Fig. 2a), which did not always correspond to the zone of release.

Of the 71 fish that were released downstream of the Big Bend rapids, 20 (28.2%) were never detected anywhere and 24 (33.8%) were only detected downstream of the rapids. Eighteen fish (25.5%) were detected somewhere in the rapids, with four of them (5.6%) being detected in the rapids for several months to over a year. The records for each of these individuals were up to 2 km apart, indicating that the fish were either dead or the transmitters were shed from the fish. Only nine fish (12.7%) were detected moving upstream of the rapids. Of the 50 fish that were released upstream of the Big Bend rapids, 9 (18%) fish were never detected anywhere and 39 (78%) were only detected upstream of the rapids. Only two fish (4%) were detected in the rapids and none were detected moving downstream of the rapids.

**Figure 3:**
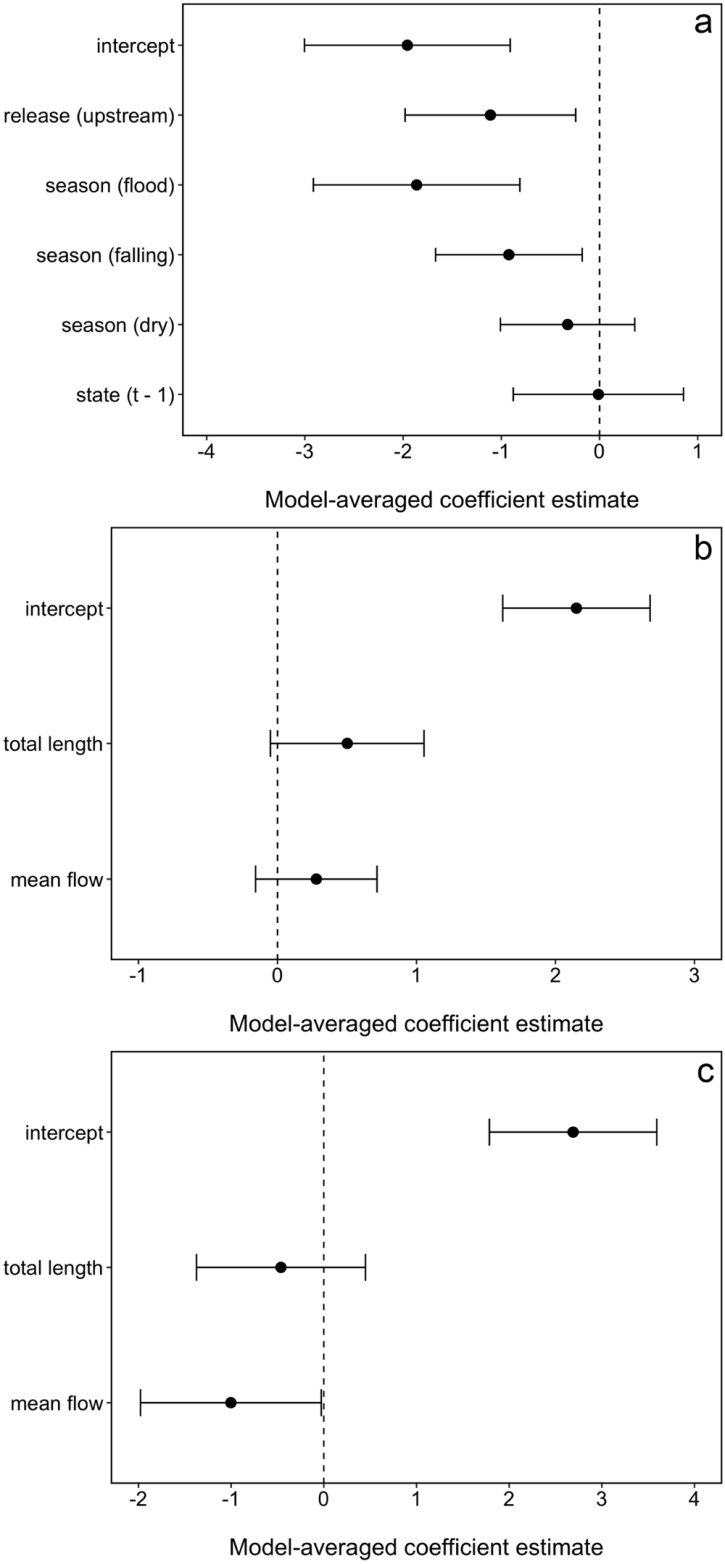
Model-averaged coefficient estimates and 95% confidence intervals for the model assessing the probability of *P. hemioliopterus* ‘moving >10 km’ upstream. (**a**) The intercept estimate refers to fish released downstream of the Big Bend rapids, not detected moving upstream at the previous month and the rising season, with all the covariates (month and fish total length) modeled with a smooth function (not shown) set at their mean values. Model-averaged coefficient estimates and 95% confidence intervals for model describing upstream (**b**) and downstream (**c**) movement rates of *P. hemioliopterus*. The intercept estimate refers to all the covariates (fish total length and mean daily flow) at their mean values. Variables were standardized before analysis.

The best approximating model describing the upstream movement of individual *P*. *hemioliopterus* in any given month included the smooth function for month and the release site (Table 2). However, the model where the smooth function for month was replaced by the effect of season had very similar support to the best approximating model (Table 2). Indeed, the 95% confidence set for the best approximating model indicated uncertainty on whether the smooth function for month or the effect of season were the best predictors of temporal variability in upstream movement (Table 2). Model-averaged fitted values indicated that *P*. *hemioliopterus* exhibited two distinct peaks in the probability of being detected moving upstream (Fig. 3). The first and higher peak occurred from November to February (end of dry season and during the rising water season), whereas the second and smaller peak occurred from May to June (falling water season).

**Table 2:** Model selection statistics for the analysis of the movement state (i.e. ‘not detected/not moving upstream’ or ‘moving upstream’) of individual *P*. *hemioliopterus* and *P*. *punctifer* in a given month.

Model	AIC_c_	∆_*m*_	*w*_*m*_	log(*L*)	*K*
*P*. *hemioliopterus*					
***f***(**month**) + **relsite**	**444**.**70**	**0**.**00**	**0**.**38**	−**217**.**31**	**5**
season + relsite	445.03	0.33	0.32	−215.44	7
*f*(month) + *f*(length) + relsite	447.13	2.43	0.11	−216.49	7
season + *f*(length) + relsite	447.64	2.94	0.09	−214.70	9
*f*(month)	449.13	4.43	0.04	−220.54	4
*f*(month) + *f*(length)	450.65	5.95	0.02	−219.27	6
*P*. *punctifer*					
***f***(**month**) + **relsite**	**164**.**89**	**0**.**00**	**0**.**40**	−**77**.**35**	**5**
*f*(month)	165.32	0.43	0.32	−78.60	4
season + relsite	168.55	3.65	0.06	−77.10	7
*f*(month) + *f*(length) + relsite	169.05	4.16	0.05	−77.35	7
*f*(month) + *f*(length)	169.25	4.36	0.04	−78.50	6
season	169.39	4.50	0.04	−78.57	6
relsite	169.84	4.95	0.03	−80.86	4

Models are ranked using the bias-corrected Akaike Information Criterion (AIC_c_) and the best approximating model for each species is shown in bold. Only the models included in the 95% confidence set for the best model of each species are shown. ∆_*m*_ is the difference in AIC_c_ units between a model *m* and the top ranked model; *w*_*m*_ is the AIC_c_ weight of model *m*; log(*L*) is the model log-likelihood; and *K* is the number of estimable parameters in the model. All fitted models included the movement state of the individuals in the previous month as a factor to account for the temporal dependence of the monthly observations; and a random effect of individual on the intercept (terms are not shown in the table). The terms *f*(month) and *f*(length) denote a smooth function for the effect of month and the fish total length (at capture) on the movement state. Release site is denoted by relsite.

**Figure 4:**
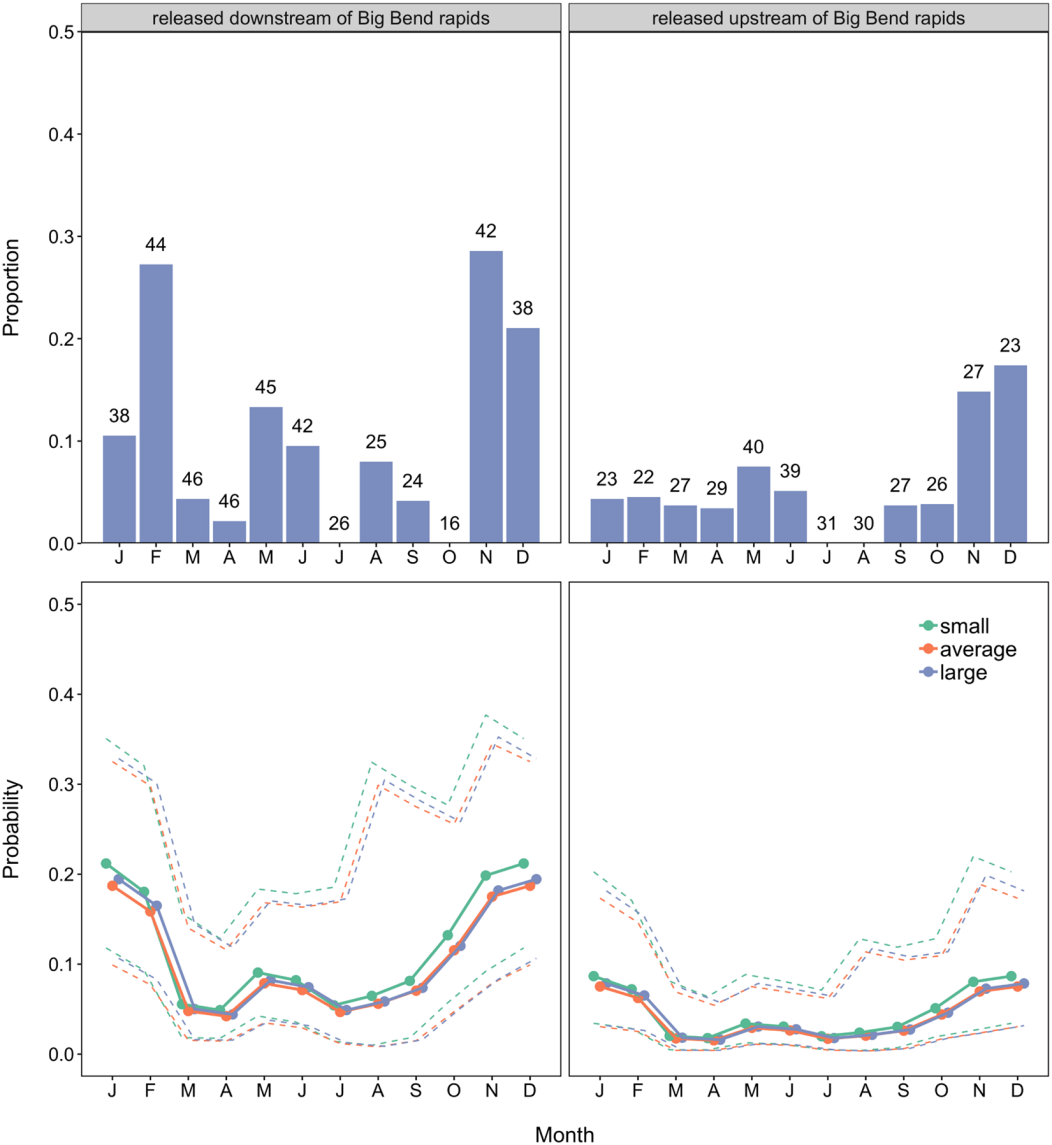
Proportion of *P. hemioliopterus* in the state ‘moving > 10 km’ upstream (top panels) and model-averaged probability (solid lines) of being detected in that movement state (bottom panels) by month (season) and release site. The numbers on top of bars in the upper panels denote the total number of *P. hemioliopterus* known to be in the monitored area that month. The dashed lines in the lower panels denote model-averaged 95% confidence intervals. Predictions were computed for an averaged size individuals (defined as the mean of total length of tagged *P. hemioliopterus*) as well as for small and large individuals (defined as mean ± 1SD of total length of tagged *P. hemioliopterus*, respectively). Predictions are only shown for fish whose state at month *t* - 1 was ‘not detected / not detected moving upstream’, as they are virtually similar to the predictions where the previous month state was ‘moving > 10 km’ upstream.

Release site was strongly supported as a predictor of upstream movement, being included in all the four top-ranked models (Table 2). In addition, the model-averaged 95% confidence interval of the coefficient estimate for release site did not overlap zero, indicating that fish released downstream of the Big Bend were about 2.8 times (95% CI: 1.1–7.7 times) more likely of being detected moving upstream than those released upstream (Fig. 3a). Model selection results also revealed some support for the smooth function of fish total length, with model-averaged fitted values indicating that small and large fish (defined as mean ± 1SD of the total length of tagged fish) were slightly more likely of being detected moving upstream than fish of average length (Fig. 4).

**Figure 5:**
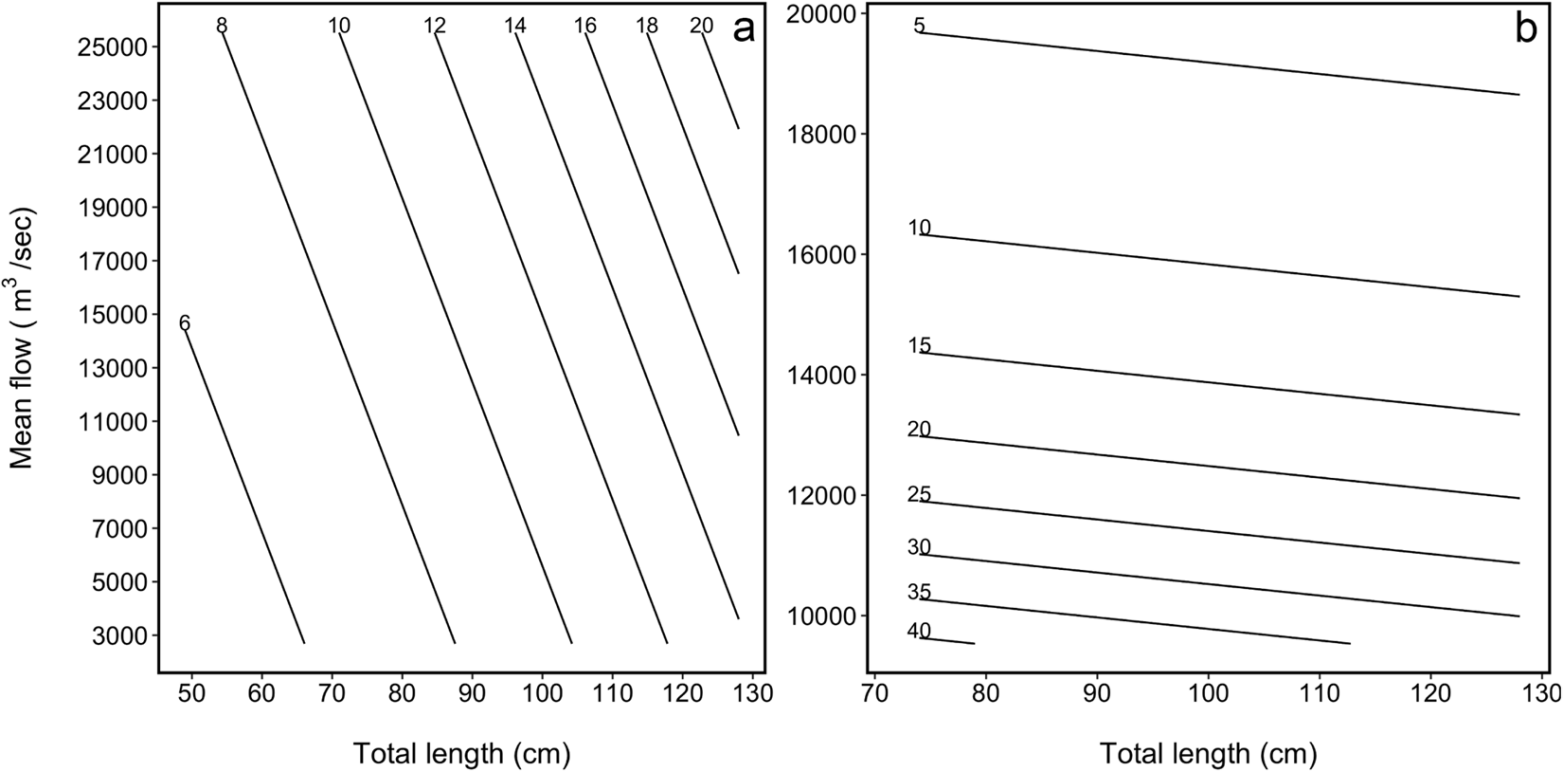
Model averaged-predictions of upstream (**a**) and downstream (**b**) movement rate of *P*. *hemioliopterus* by total length and mean daily flow. Values on top of isolines denote predicted movement rate (km/day) for a specific combination of fish total length and mean daily flow.

Among the *P*. *hemioliopterus* detected moving >10 km, the median (minimum–maximum) distances moved were 37 km (12–347 km) upstream (Fig. 2b) and 25 km (13–128 km) downstream. The median (minimum–maximum) movement rates computed between fixed receivers were 10.4 km/day (0.7–70.7 km/day) upstream and 30.5 km/day (0.3–81.4 km/day) downstream. The best approximating model describing upstream movement rates included both fish total length and mean flow (Table 3). Yet, there was substantial uncertainty in model selection, with the models including only length or no effects having similar support from the data than the top-ranked one. The model-averaged coefficients and fitted values revealed that upstream movement rates were positively associated with both length and mean flow (Figs 3b and 5a), though the 95% confidence interval for the model-averaged coefficients overlapped zero (Fig. 3b).

**Table 3:** Model selection statistics for the analysis of the movement rate of individual *P*. *hemioliopterus* and *P*. *punctifer* in a given month.

Model	AIC_c_	∆_*m*_	*w*_*m*_	log(*L*)	*K*
*P*. *hemioliopterus* (upstream)					
**length** + **mflow**	**98**.**95**	**0**.**00**	**0**.**31**	−**43**.**36**	**5**
length	98.98	0.03	0.31	−44.77	4
no effects	99.32	0.37	0.26	−46.24	3
mflow	100.98	2.04	0.11	−45.78	4
*P*. *hemioliopterus* (downstream)					
**mflow**	**58**.**90**	**0**.**00**	**0**.**63**	−**23**.**23**	**4**
no effects	61.24	2.34	0.20	−26.42	3
length + mflow	62.12	3.22	0.13	−22.31	5
*P*. *punctifer* (upstream)					
**no effects**	**49**.**49**	**0**.**00**	**0**.**78**	−**20**.**41**	**3**
mflow	53.39	3.90	0.11	−20.19	4
length	53.70	4.21	0.10	−20.35	4

Models are ranked using the bias-corrected Akaike Information Criterion (AIC_c_) and the best approximating model for each species is shown in bold. Only the models included in the 95% confidence set for the best model of each species are shown. ∆_*m*_ is the difference in AIC_c_ units between a model *m* and the top ranked model; *w*_*m*_ is the AIC_c_ weight of model *m*; log(*L*) is the model log-likelihood; and *K* is the number of estimable parameters in the model. All fitted models included a random effect of individual on the intercept (term is not shown in the table). The term mflow denotes mean daily flow.

As for downstream movement rates, the best approximating model included only the effect of mean flow (Table 3). This model had about three times more support than the second ranked model that did not include any effects. The top ranked model also had about five times more support than the model including both length and mean flow (Table 3). The model-averaged coefficient for mean flow was negative and its 95% confidence interval did not overlap zero (Fig. 3c). The model-averaged coefficient for length was also negative but its 95% confidence interval did overlap zero (Fig. 3c). Model-averaged predictions indicated that downstream movement rates increased with decreases in mean flow and decreased only slightly with increasing length (Fig. 5b).

### *Pseudoplatystoma punctifer*

A total of 57 (93.4% of 61 tagged) *P*. *punctifer* were detected after release. Thirty fish (52.6% of 57) were always detected in the same zone where they were released. Individual detections from nine (15.8%) of these fish always occurred within 2 km, suggesting that the fish were either dead or the transmitters were shed from the fish. Twenty-three fish (40.4%) made unidirectional movements between zones, with five (8.8%) and 18 (31.6%) fish moving downstream and upstream of the release zone, respectively. Four fish (7%) made bidirectional movements between zones, with one fish (1.8%) first moving downstream and then upstream across zones; two fish (3.5%) first moving upstream and then downstream across zones; and one fish (1.8%) repeatedly moving downstream and upstream between adjacent zones. Except for this latter case, the fish returned to the same zone from where they were released.

Of the 40 fish that were released downstream of the Big Bend rapids, one (2.5%) was never detected anywhere and 23 (57.5%) were only detected downstream of the rapids. Ten fish (25%) were detected somewhere in the rapids, with two (5%) being detected in the area for nearly one year. The records for each of these two individuals were up to 2 km apart, indicating that the fish were either dead or the transmitters shed from the fish. Only six fish (15%) were detected moving upstream of the rapids. Of the 21 fish that were released upstream of the Big Bend rapids (13 at zone 4 and eight at zone 6), two were never detected anywhere (9.5%) and 17 (81%) were only detected upstream of the rapids. Only two fish (9.5%) were detected in the rapids, both of which were released at zone 4 and none were detected moving downstream of the rapids.

**Figure 6:**
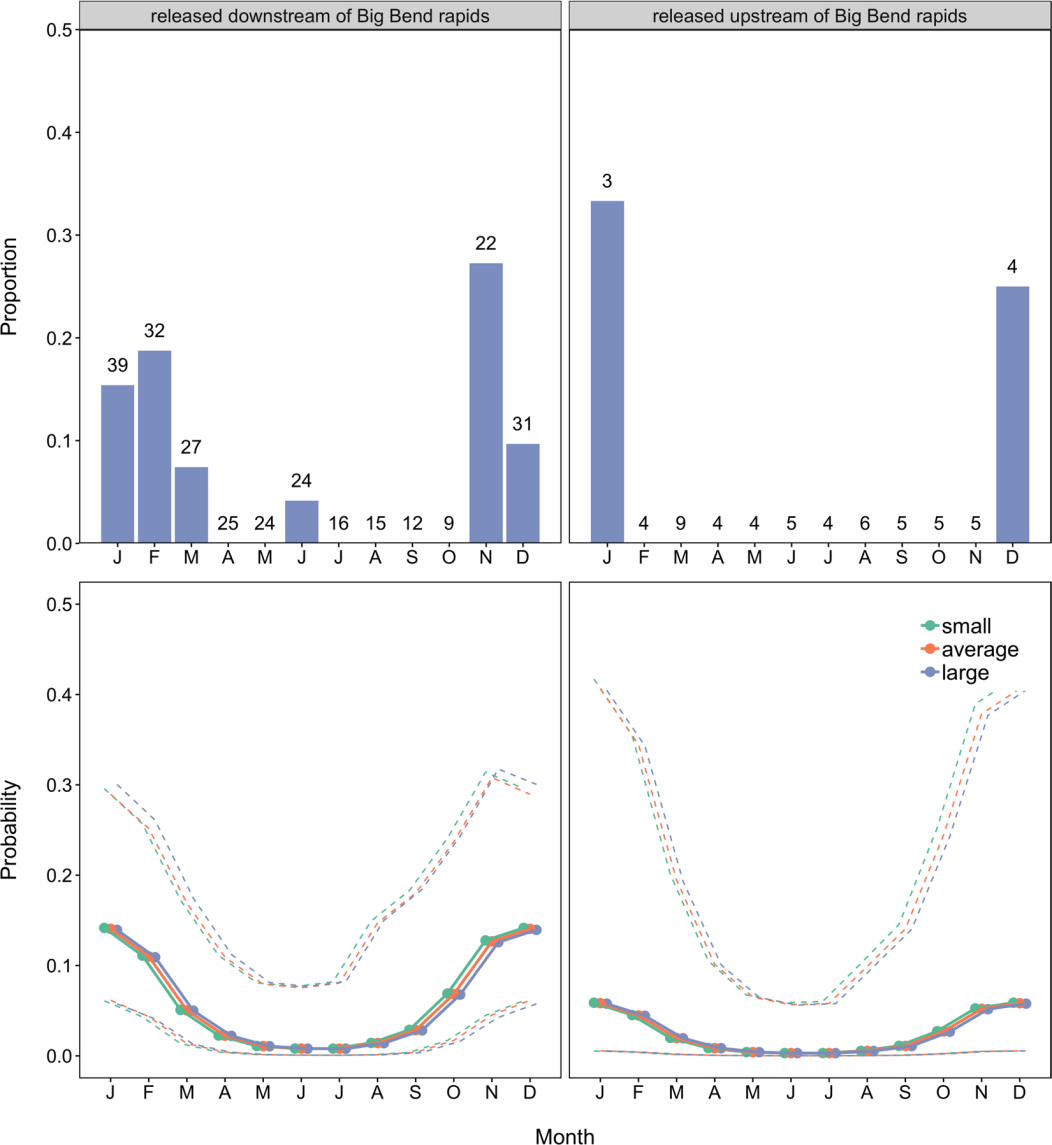
Proportion of *P*. *punctifer* in the state ‘moving >10 km’ upstream (top panels) and model-averaged probability (solid lines) of being detected in that movement state (bottom panels) by month (season) and release site. The numbers on top of bars in the upper panels denote the total number of *P*. *punctifer* known to be in the monitored area that month. The dashed lines in the lower panels denote model-averaged 95% confidence intervals. Predictions were computed for an averaged size individuals (defined as the mean of total length of tagged *P*. *punctifer*) as well as for small and large individuals (defined as mean ± 1SD of total length of tagged *P*. *punctifer*, respectively). Predictions are only shown for fish whose state at month *t* − 1 was ‘not detected/not detected moving upstream’, as they are virtually similar to the predictions where the previous month state was ‘moving >10 km’ upstream.

Similarly to *P*. *hemiolopterus*, the best approximating model describing the upstream movement of individual *P*. *punctifer* in any given month included the smooth function for month and the release site (Table 2). However, the 95% confidence set for the best approximating model also indicated uncertainty on whether the smooth function for month or the effect of season were the best predictors of temporal variability in upstream movement (Table 2). Only one of the seven models included in the 95% confidence set did not include either the smooth function for month or the effect of season, but that model had little support from the data (Table 2). Model-averaged fitted values indicated a single peak, between November and February (i.e. end of dry season and during the rising water season), in the probability of detecting *P*. *punctifer* moving upstream (Fig. 6).

**Figure 7:**
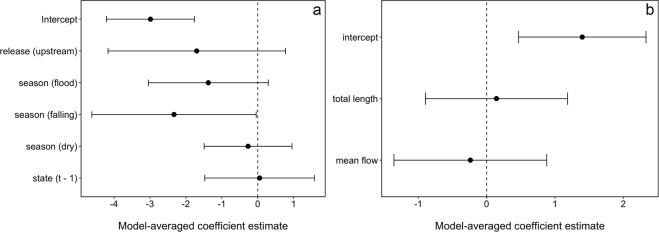
Model-averaged coefficient estimates and 95% confidence intervals for model assessing the probability of *P*. *punctifer* ‘moving >10 km’ upstream. (**a**) The intercept estimate refers to fish released downstream of the Big Bend rapids, not detected moving upstream at the previous month and the rising season, with all the covariates (month and fish total length) modeled with a smooth function (not shown) set at their mean values. Model-averaged coefficient estimates and 95% confidence intervals for model describing upstream movement rates of *P*. *punctifer*. (**b**) The intercept estimate refers to all the covariates (fish total length and mean daily flow) at their mean values. Variables were standardized before analysis.

Although release site was included in the best approximating model, model selection indicated substantial uncertainty about its effect. Nearly half of the models in the 95% confidence set did not have the effect of release site, including the second ranked model, which had very similar support to the best approximating model (Table 2). Indeed, although model-averaged fitted values indicated that fish released upstream of the Big Bend were less likely of being detected moving upstream, the coefficient for this effect and the associated predictions exhibited high uncertainty (Figs 6 and 7a). Model selection results also revealed some support for the smooth function of fish total length, with model-averaged fitted values indicating a minor decrease in the probability of being detected moving upstream with increases in size (Fig. 6).

**Figure 8:**
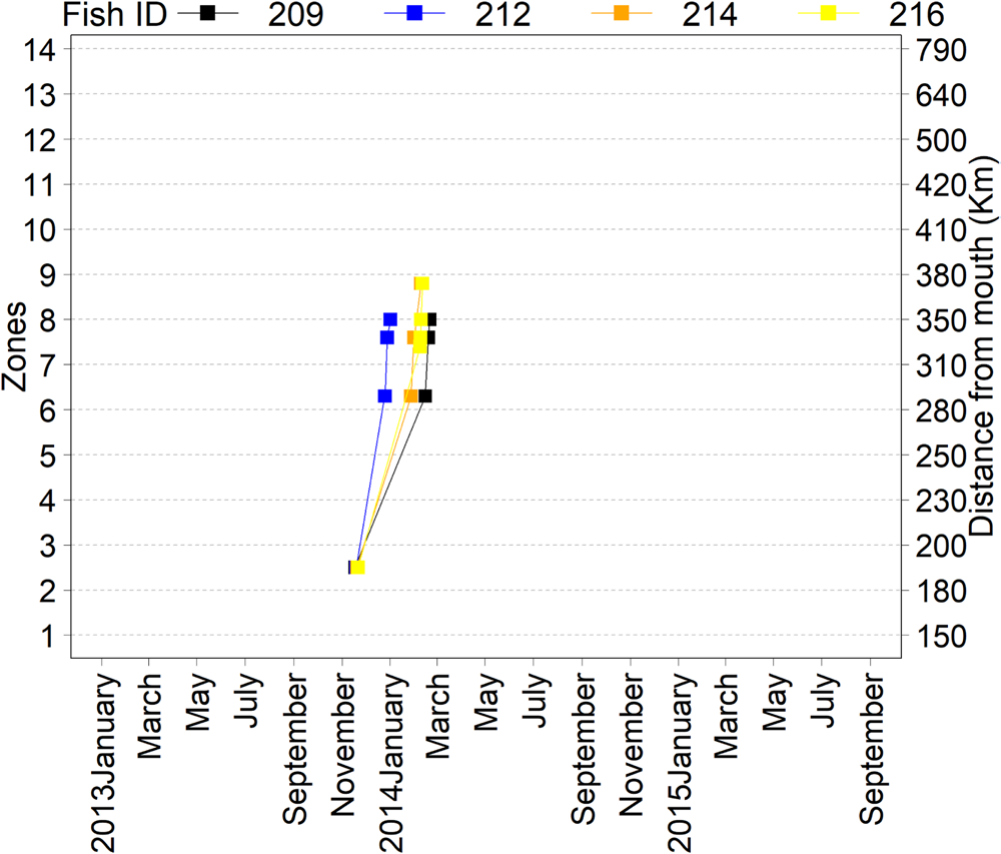
Synchronized movements of four individuals of *P*. *punctifer*.

Among the *P*. *punctifer* detected moving >10 km, the median (minimum–maximum) distances moved were 25 km (11–164 km) and 20 km (10–63 km) upstream and downstream, respectively. The median (minimum–maximum) movement rates computed between fixed receivers were 5.1 km/day (0.4–23.3 km/day) upstream and 24.1 km/day (19.9–28.3 km/day) downstream. The best approximating model describing upstream movement rates did not include either length or mean flow (Table 3). In fact, the models including any or both of these effects had very little support from the data compared to the model without any effects (Table 2). Consequently, model-averaged coefficients were close to zero and their 95% confidence interval overlapped zero (Fig. 7b). Four tagged *P. punctifer* released downstream of the Big Bend in November 2013 moved to an area located 70 km upstream of zone 4, arriving between January-February 2014 (Fig. 8).

## Discussion

Investigating fish migrations in freshwater is a difficult task due to the challenges (i.e. turbidity, water depth and velocity) associated with conducting direct observations or inability to track organisms underwater with GPS and other satellite-based technologies^24^. These challenges are exacerbated by the fact that fish migrations often occur over large distances^25,26^ and through a variety of complex habitats^27–29^. It is especially true in large Amazonian rivers, such as the Xingu River, which are characterized by large dimensions (river length ranging from hundreds to thousands of kilometers and widths up to 14 km^21^), habitat complexity (braided channels, deep pools, lateral flooded areas covered by dense vegetation), extreme seasonal water level variation (over 10 m) and difficult access. Considering these challenges, the percentage of tagged fish detected in this study moving beyond the release site, and often for long distances, can be considered satisfactory (i.e., *P*. *hemioliopterus* = 56.6% and *P*. *punctifer* = 47.4%). It is possible that some fish were not recorded moving away from the release site due to fishing. A total of seven tagged fish (*P*. *hemioliopterus* = 5, *P*. *punctifer* = 2) were recaptured and reported by fishers in the Xingu River within 90 days from being released^30^. In the São Francisco River, up to 62.5% of radio-tagged *Pseudoplatystoma corruscans* were potentially harvested by fishers^31^. Loss of tagged fish by harvest is common and often higher than 30%^32^. Also, it is possible that the migratory movements exhibited by some tagged fish were interrupted by their harvest, which would result in underestimates of the migration distances reported here.

The relatively high percentage of tagged *P*. *hemioliopterus* and *P*. *punctifer* always detected in the released zone in this study (43.5% and 52.6%, respectively) could be associated to mortality or transmitter loss. We have no experimental data on mortality or transmitter loss for the study species, but transmitter loss has been reported for other catfish species in experimental conditions at a rate of 30% for *Rhamdia quelen* and 53.3% for *Ictalurus furcatus*^33,34^. Alternatively, the high percentage of fish that was always detected in the release zone could be associated with the occurrence of resident individuals, suggesting that *P*. *hemioliopterus* and *P*. *punctifer* might exhibit partial migration. Partial migration, where populations are composed of both migratory and resident individuals^35^, is well known for groups such as salmonids and has been documented in various fish orders, though there is little evidence that it occurs in catfish^25,26^. Furthermore, this study also revealed that some *P*. *hemioliopterus* remained resident in one year and migrated in another, which is another form of partial migration, and may represent skipped spawning in years when fish remained resident^36^. This form of partial migration was also observed in females of another Neotropical catfish, *P*. *corruscans*, in the São Francisco River^31^. Determining whether partial migration in the study species is associated with skipped spawning will require the implementation or development of approaches to confidently sex and determine the maturation status of fish being tagged for tracking. Obtaining information on maturation status would also enable one to determine if a fish’s decision to not migrate might be a stress response to capture and tagging (e.g. a fish deemed mature at tagging but not migrating to spawning areas).

*Phractocephalus hemioliopterus* and *P*. *punctifer* were mainly detected moving upstream during the end of dry/rising period, with a second smaller peak for *P*. *hemioliopterus* occurring in the falling period. In the Colombian Amazon (Caquetá River) upstream movements have been recorded for the two species during the rising season, with another peak in the dry season for *P*. *hemioliopterus*^37,38^. More generally, upstream movements for spawning are well documented for Neotropical migratory fishes and their timing is associated with the onset of the floods^39,40^. Reaching the spawning areas before the floods is thought to be critical to enable eggs and early life stages to take advantage of higher river levels and water velocities while drifting downstream, which increases the chances of accessing rearing habitats in the flooded areas downstream^27,39^. However, critical habitats (spawning, rearing) for *P*. *hemioliopterus* and *P*. *punctifer* in the Xingu River remain unknown. Another characteristic of upstream spawning migration of Neotropical fishes is that they are typically followed by a downstream return migration to feeding areas^27,31,39^. Indeed, several *P*. *hemioliopterus* and *P*. *punctifer* performed bidirectional long-distance movements in the Xingu River. Furthermore, among the *P*. *hemioliopterus* released upstream of the Big Bend, some individuals returned to the departure zone after having moved long distances (>100 km) upstream.

The movement rates varied substantially between *P*. *hemioliopterus* and *P*. *punctifer* and even between individuals within the same species. *Phractocephalus hemioliopterus* moved upstream at a rate twice as fast as that recorded for *P*. *punctifer*. To our knowledge, this is the first time that movement rates have been recorded for *P*. *hemioliopterus*. The upstream movement rates recorded for the *P*. *punctifer* in this study (0.4 to 23.3 km day^−1^) were similar to those recorded for one of its congeners, *P*. *corruscans*, in the São Francisco River (1.9 to 29.1 km day^−1^)^31^, though they were lower and less variable (0.9 to 3.6 km day^−1^) in the Paraná River^41,42^. For both species (*P*. *hemioliopterus* and *P*. *punctifer*) in this study, downstream movement rates were higher than upstream rates. The slower upstream movements could be related to the presence or rapids in most of the Xingu River in study area, with higher water velocities imposing a reduction in the upstream speed of fish movements and on the other hand facilitating the downstream movements.

The maximum distance recorded for *P*. *hemioliopterus* in this study (347 km) is very short in comparison to that traveled by other large pimelodids, such as the species of the genus *Brachyplatystoma* (i.e., 3,129 km to 5,786 km), in the Amazon^4^. However, the distances travelled by *P*. *hemioliopterus* in the Xingu River can be considered one of the longest ever recorded for large catfish, other than *Brachyplatystoma*, in Neotropical rivers. Based on the accounts of fishers, *P*. *hemioliopterus* was previously classified as a medium distance migratory species^43^ (100–500 km) and the distances recorded by telemetry in this study support this classification. Moreover, the detections of four fish in the most upstream fixed station (zone 13) with no records of return within the life time of transmitters, suggests that *P*. *hemioliopterus* could have moved further upstream the study area.

Migratory behaviour of large catfish of the genus *Pseudoplatystoma* is better known than that of *P*. *hemioliopterus* and has been described for populations from various rivers in South America^31,39,41^. In the Colombian Amazon, it is assumed that *Pseudoplatystoma* spp. migrates between 300–500 km^38^, but no movement data are available. In our study, the longest distances recorded for *P*. *punctifer* (164 km) were shorter compared to that recorded for other *Pseudoplatystoma* species outside the Amazon Basin (in rivers from Midwest to Southern Brazil). A radio-tagged *P*. *corruscans* in the Paraná River was recaptured by a fisher 400 km upstream of the release site^42^, and in the São Francisco River the same species was detected moving 274 km along the mainstem^31^. The relatively shorter movements observed for *P*. *punctifer* in this study could be related to the proximity of the release sites (zones 2 and 6) to the location of a potential spawning site (see below) for the species just upstream of the Big Bend.

Rapids and waterfalls can represent a barrier to the movements and dispersal of fish populations in Amazonian rivers^44,45^. Despite the rapids in the Big Bend being a natural biogeographic barrier affecting the distribution of fish^45,46^, the studied species were detected moving upstream into or past this obstacle. Furthermore, data analyses indicated that *P*. *hemioliopterus* and *P*. *punctifer* released downstream of the Big Bend had greater probability of exhibiting upstream movements, compared with those released upstream of the bend. The higher probability to move upstream for the fish released downstream of the Big Bend suggests that some of the spawning grounds used by the population of tagged *P*. *hemioliopterus* and *P*. *punctifer* may be located upstream of the rapids. Indeed, local fishers report that important spawning grounds for *P*. *punctifer* occur in areas located at zone 8 (70 km upstream the upper limits of the Big Bend). Interestingly, four tagged *P*. *punctifer* released downstream of the Big Bend in November 2013 moved upstream to this supposed spawning area. Additionally, eight tagged *P*. *punctifer* captured and released in this area were never recorded upstream. Four of them were recorded just in the release site and four moved downstream. In the Upper Amazon, *P*. *punctifer* seems to spawn in a variety of habitats, including headwater streams and deep pools^47^. Future targeted studies with larger number of tagged fish and eggs and larvae sampling could determine if the area upstream of the Big Bend is a critical spawning site for *P*. *punctifer* in the Xingu River.

The possibility that *P*. *hemioliopterus* and *P*. *punctifer* migrate upstream of the rapids to access their spawning grounds highlights the need to ensure that the fishway built in the Pimental site of the BMHC be monitored for its effectiveness in attracting and enabling the passage of these species. However, it is still unknown if the reduction in the river discharge on the stretch of the Xingu River downstream of the Pimental site (zones 2–6) is restricting or even blocking the migration of large catfish in the area. Before completion of the dam, flow varied drastically between dry and flood season (700 to 26,000 m^3^/s). According to the “Integrated Management Plan for the Big Bend”, the BMHC should keep flows between 700 to 8,000 m^3^/s^48^. Although the flows in the dry season remain like that before the completion of the dam, the maximum flows in the flood season were drastically reduced. In general, there is much uncertainty about how reductions in flow caused by hydroelectric dams will affect fish populations in the Amazon^45^.

Despite the increasing number of telemetry studies in Brazil since 2001, this was the first large scale telemetry study in a river in the Amazon basin. The results gathered, despite the challenges associated with access and extension of the study area, proved that telemetry can be successfully used to investigate fish movements in an Amazon scale. Future studies coupling telemetry with different techniques, such as genetics and physiology, will improve our understanding about fish movements^49,50^ and will allow us to draw the fish migration maps, which are very important for sustainable management of fisheries^28^, in large Amazonian rivers affected by dams.

The operation of BMHC and its fish passage facility (which started to operate in 2016, after the end of this study) as a tool to mitigate the impacts on migratory species is directly dependent on the knowledge of basic aspects of fish migration before the dam construction. Before this study, the information on the movements of *P*. *hemioliopterus* and *P*. *punctifer* in the Xingu River were just anecdotal. The findings presented here addressed basic questions about the movements of large catfish species in the Xingu River (i.e., capacity to migrate through the Big Bend rapids, seasonality of movements, traveled distances), and will likely prove critical for the effective management of these species. We demonstrated in this study that migratory fish were able to overcome the rapids of the Big Bend before the river impoundment. Although fish ladders have proven ineffective as a conservation strategy for many migratory Neotropical fish species^14^, it could be the only option for the migratory catfish in the population to reach purported spawning areas upstream of the Pimental site. Long term monitoring will be critical to identify if the migratory movements exhibited by the populations in the pre-impoundment phase will continue through the post-impoundment phase with much reduced flows.

## Methods

### Study area

We conducted the study along 685 km of the lower and middle Xingu River, in the Brazilian Amazon. The study area also included the lower reaches (about 3 km) of two large tributaries to the Xingu River, the Iriri and Bacajá Rivers. The study area was divided into 14 zones based on the characteristics of the river segments (i.e. occurrence of rapids, deep pools, sandy or rocky bottom), the limits of the Big Bend, the location of BMHC sites and the river extension and tributaries (Fig. 1).

The Iriri and the Bacajá Rivers are two of the main tributaries to the lower and middle Xingu River. The Iriri River is also a clearwater river entering the Xingu River about 150 km upstream of the Big Bend. This river is very similar to the Xingu River in that it has rocky bottom and numerous rapids and waterfalls, which are also thought to function as natural barriers for many aquatic organisms. The Bacajá River flows into the Big Bend, draining water from a large rocky area that is characterized by high particle levels, in contrast to the Xingu and Iriri waters^19^. Both the Iriri and Bacajá Rivers are considered important areas for rheophilic species such as large catfish and characiform fishes. The limits of the Big Bend are located between zone 3 (downstream; 3°7′28.60″S 51°42′0.12″O) and zone 7 (upstream; 3°12′58.38″S 52°12′30.58″O) but the majority of the rapids are located in zones 3 and 4.

### Fish sampling and tagging

We captured fish in February, March and November 2013 and January and February 2014 at four sites in the Xingu River (two upstream and two downstream of the Big Bend rapids) using longline fishing and hand line. Captured fish were immediately put into tanks (500 L) filled with river water and transported by boat to the tagging site, where they were transferred to holding tanks (1,000–2,000 L) for recovery (5–30 min) prior to tagging. Fish were then anesthetized by immersion in a bath of clove oil (eugenol dissolved in ethyl alcohol) diluted in river water (10 ml of eugenol in 500 L water). Immediately after losing equilibrium and exhibiting little ventilation (stage II-2)^51^, we measured total length (TL, cm) and mass (kg), and transferred fish to a surgery sling, where they were kept under forced gill ventilation and anesthesia (2 ml of eugenol per 100 L of water). We fitted each individual with a combined acoustic and radio transmitter (Lotek Wireless CART Series®, models MM-MC-16-50 [16 × 86 mm, 37 g in the air, life of 531 days at a 5 sec burst rate for radio and 10 sec for acoustics] and MM-MC-16-33 [16 × 73 mm, 31 g in the air, life of 332 days at a 5 sec burst rate for radio and 10 sec for acoustics], which was surgically implanted into the abdominal cavity^52^. In addition to the transmitters, we marked fish with external hydrostatic anchor tags (Hallprint©, model standard TBA) to facilitate identification and return of transmitters and/or tagged fish recaptured by fishers^30^. The duration of surgery was 2–15 min (median 6 min). After surgery, we transferred the individuals to a holding tank (1000–2000 L) with a continuous supply of river water and monitored them for three to four hours before release. We then released fish in the river near the tagging site. Fish capture, handling and tagging procedures were carried out in accordance with the laws of Brazil and the experimental protocol was approved by the Brazilian Institute of the Environment – IBAMA (Permit Number 145/2012).

### Telemetry equipment and fish monitoring

Due to the high environmental heterogeneity in the study area, varying from shallow (less than two meters) to deep waters (over 80 m) with rocky and sandy bottoms^20^, we used a combination of radio and acoustic telemetry to continuously monitor the tagged fish. We deployed acoustic and radio receivers along 500 km of the study area in nine of the 14 established zones (Fig. 1). We installed acoustic receivers (Lotek WHS 3250) in seven zones using gate arrays with estimated detection range between 600–800 m (2–4 receivers per zone, with an estimated overlap of detection of at least 100 m), except in zone 10 (Iriri River), where we deployed only one acoustic receiver due to the narrow width of the river (up to 800 m in the flood season in this zone). Receivers were attached to a system composed by moorings, steel cables and floats, designed to adjust to water level changes throughout the hydrological cycle. We placed radio receivers (Lotek Wireless SRX-DL and SRX 600) connected to Yagi antennas with three to five elements (2–4 per station, AV Antronics) in steel towers (10 m high) located on the river banks in nine of the 14 established zones (Fig. 1). The estimated detection range of radio signals was between 800–1,000 m. Additionally, we tracked fish by boat and airplane to complement the passive monitoring and to detect fish in areas not covered by fixed stations. We conducted tracking by boat monthly from July 2013 to July 2015 (total of 25 boat transects; 392 hours); and aerial tracking in May 2013, November 2013, February 2014, June 2014 and November 2014 (total of five flights; 26 hours). In both situations, we performed tracking during the day with a radio receiver (Lotek Wireless SRX 600) equipped with three-element Yagi antenna and an internal GPS, which recorded the position of every fish when a signal was received.

### Data processing

We processed raw acoustic and radio detection data obtained by passive monitoring to keep only valid detection records^53^. The radio detections obtained by mobile tracking were assessed during the tracking based on power thresholds and multiple detections that were consistent with the tag transmission rate (only detections deemed valid were retained). We then determined ‘detection events’ (also known as ‘residence events’) for each fish. A detection event consisted of a sequence of detections in the same zone and was terminated when: (i) the time elapsed between any two consecutive detections in the same zone was >30 days; or (ii) the fish was detected in another zone. We considered detections obtained during mobile tracking as individual detection events.

We computed the shortest within-river distances moved between detection events for each fish and used these distances to determine whether the fish had: (i) not moved or remained resident around the same area (i.e. distances moved were <10 km); or (ii) moved >10 km upstream or downstream. To assess the probability of fish moving >10 km upstream or downstream (see *Statistical modeling of movement events*), we created two binary time-series (one each for upstream and downstream movements) at monthly steps for each individual, ranging from the first to the last month when the fish was detected. We assigned each month of the time series a value of 1 if the fish had been detected moving >10 km; or a value of 0 if a fish had not moved >10 km or had being detected moving >10 km in the opposite direction or had not being detected. To compute movement rates (km/day) for upstream and downstream movements, we considered only detections obtained by the fixed acoustic and radio receivers. This was done due to the biases in movement rate estimates that would be caused by the fact that the time of a mobile detection could occur much later than the time when a fish would have arrived at a location or much earlier than the time it would have left the location.

#### Statistical modeling of movement events

We analyzed the binary time-series of monthly movements >10 km separately for each species by treating movements as a first-order Markov process^54^. This approach was used because such movements are likely to be correlated over time^55^. For example, an individual embarking on a long-distance movement may be more likely to be detected moving >10 km in one direction in a given month if it was also detected moving >10 km in the same direction in the previous month. Conversely, if the long-distance movement occurs within a month, then one may expect that an individual detected moving more >10 km in a given month is less likely to still be making such movements in the next month. Defining *S*_*t*_ as a binary random variable denoting the state–(1) moving >10 km or (0) otherwise–of an individual at month *t*, the model can be written as

1 $$\begin{array}{c}\mathrm{log}\,{\rm{it}}({s}_{i,t})=(\alpha +{\gamma }_{i})+{\beta }_{1}{x}_{1,i,t}+\cdots +{\beta }_{k}{x}_{k,i,t}+{\beta }_{s}{s}_{i,t-1}\,\,\\ \,{\gamma }_{i}\,\sim \,N(0,{\sigma }_{\gamma }^{2})\end{array}$$

where *s*_*i*,*t*_ and *s*_*i*,*t*−1_ are, respectively, the movement state of individual *i* at month *t* and *t* − 1; *α* is the intercept; *γ*_*i*_ is the individual-specific deviation from the intercept, which is assumed to be normally distributed with mean zero and variance ${\sigma }_{\gamma }^{2}$; *β*_*k*_ for *k* = {1 … *k*} are the coefficients describing the effect of the covariate *x*_*k*,*i*,*t*_; and *β*_*s*_ is the coefficient describing the effect of the previous movement state of the individuals.

We included the following covariates in the analysis: (i) the total length of the fish at capture (numeric variable in cm); (ii) the release site (factor variable with two levels: upstream and downstream of the Big Bend rapids); (iii) the month (numeric variable); (iv) the season based on the flood cycle (factor variable with four levels: rising [December to February], flood [March to April], falling [May to July] and dry [August to November]); (v) mean monthly rainfall (numeric variable in mm); and (vi) mean monthly flow (numeric variable in m^3^/s). Monthly flow and rainfall data were provided by LEME Engenharia. Due to the potential non-linearities in the relationship between an individual movement state and total length, month, mean rainfall and mean flow, we modeled these variables using smooth functions that were estimated using generalized additive mixed models (GAMMs). The variable month was modeled with a cyclic regression spline, which is appropriate for cyclic or seasonal relationships, whereas all the other variables were modeled with a cubic regression spline^56^. However, due to the close relationship between month, season, mean rainfall and mean flow, we did not include these variables in the same model to avoid issues arising from multicollinearity^57^. Instead, we fitted four separate sets of models including only one of these time-dependent variables in addition to subsets of the other covariates. Each set included models with the following combination of variables: (1) intercept-only; (2) length; (3) release site; (4) *time*; (5) length + release site; (6) length + *time*; (7) release site + *time*; (8) length + release site + *time*; where *time* refers to one of the four time-dependent variables (month, season, mean rainfall or mean flow). Since models excluding *time* do not differ among the four subsets of models, a total of 20 models were fitted for each species. The variable denoting the fish movement state at month *t* − 1 was kept in all models. The model intercept was allowed to vary with individual by including fish ID as a random factor.

We conducted the analysis only for upstream movements because downstream movements were not detected enough to warrant adequate analysis using our modeling framework (i.e. most of the time-series of downstream movements consisted of zeros). All numeric variables were standardized before analysis by subtracting the mean and dividing the result by the variable standard deviation. We pooled data from all years due to insufficient sample sizes to estimate the effect of year.

#### Statistical modeling of movement rates

We analyzed movement rates separately for each species using linear mixed models to account for repeated observations of the same individuals^57,58^. The model can be written as

2 $$\begin{array}{c}\mathrm{log}\,({m}_{i,t})=(\alpha +{\gamma }_{i})+{\beta }_{1}{x}_{1,i,t}+\cdots +{\beta }_{k}{x}_{k,i,t}\,\\ \,{\gamma }_{i}\,\sim \,N(0,{\sigma }_{\gamma }^{2})\end{array}$$

where *m*_*i*,*t*_ is the movement rate of individual *i* at time *t*; *α* is the intercept; *γ*_*i*_ is the individual-specific deviation from the intercept, which is assumed to be normally distributed with mean zero and variance ${\sigma }_{\gamma }^{2}$; and *β*_*k*_ for *k* = {1 … k} are the coefficients describing the effect of the covariate *x*_*k*,*i*,*t*_. We included the following covariates in the analysis: (i) the total length of the fish at capture (numeric variable in cm) and (ii) mean daily flow in the month a given movement event was initiated (numeric variable in m^3^/s). Due to the small sample sizes, we did not include interactions between length and mean flow and did not consider using smooth functions for these variables. Both length and mean daily flow were standardized before analysis by subtracting the mean and dividing the result by the variable standard deviation. We fitted a total of four models in the analysis for each species.

The model intercept was allowed to vary with individual by including fish ID as a random factor. Although the nature of the data suggests the potential for temporal correlation in the residuals, model comparisons based on Akaike Information Criterion adjusted for biases caused by small sample sizes (AICc)^59^ (see next section) did not indicate the need to incorporate a correlation structure in the analysis. We log-transformed movement rates before the analysis to constrain model predictions to be non-negative. We conducted the analysis separately for upstream and downstream movement rates of *P*. *hemioliopterus*. For *P*. *punctifer*, we conducted the analysis just for upstream movement rates, as only two observations of downstream movement rates were available. We pooled data from all years due to insufficient sample sizes to estimate the effect of year.

#### Model checking, selection and averaging

We checked the models graphically and considered them valid if no obvious patterns were present in the Pearson and deviance residuals plotted against fitted values and the covariates^57^. We checked the assumption of normality of individual random effects using QQ-plots^57^. However, we note that the small sample size in some cases (e.g. movement rates) prevented us from confidently assessing the model assumptions. We fitted the models to the data using packages ‘gamm4’^60^ or ‘nlme’^61^ in R 3.2^59^.

We conducted model selection using AICc. The best approximating model describing variability in the data was identified as the one with the lowest AICc value. We used differences in AICc values (Δ_*m*_) between model *m* and the best approximating model to calculate the AICc weights (*w*_*m*_) of the models. To account for model selection uncertainty, we calculated model-averaged estimates of model coefficients, predictions and associated variances based on the 95% confidence set for the best approximating model (i.e. models included in a set where the sum of *w*_*m*_ from largest to smallest is just ≥ 0.95)^62^. We used the ‘natural average’ method to average the model coefficients^63^. Model selection was conducted using package ‘MuMIn’^64^ in R 3.2^61^.

### Ethical statement

Herewith the authors declare the study was performed in accordance with the Brazilian laws and protocols were approved by the Brazilian Institute of the Environment – IBAMA (Permit Number 145/2012).

## Data Availability

The datasets generated during and/or analyzed during the current study are available from the corresponding author upon request.
